# 2-[(2,4-Dimethyl­phen­yl)imino­meth­yl]-3,5-dimethoxy­phenol

**DOI:** 10.1107/S1600536809021278

**Published:** 2009-06-13

**Authors:** Hasan Tanak, Yelda Bingöl Alpaslan, Metin Yavuz, Erbil Ağar, Ferda Erşahin, Orhan Büyükgüngör

**Affiliations:** a Department of Physics, Faculty of Arts & Science, Ondokuz Mayıs University, TR-55139 Kurupelit–Samsun, Turkey; b Department of Chemistry, Faculty of Arts & Science, Ondokuz Mayıs University, 55139 Samsun, Turkey

## Abstract

X-ray analysis reveals that the title Schiff base compound, C_17_H_19_NO_3_, possesses both OH and NH tautomeric character in its mol­ecular structure. The occupancies of the enol and keto tautomers are 0.62 (3) and 0.38 (3), respectively. The presence of the minor keto form could not be confirmed from the IR spectrum. The mol­ecule is approximately planar, the dihedral angle between the planes of the two aromatic rings being 6.97 (8)°. The mol­ecular structure of the major component is stabilized by an intra­molecular O—H⋯N hydrogen bond, which generates an *S*(6) ring motif (N—H⋯O hydrogen bond in the minor component).

## Related literature

For tautomeric forms of Schiff bases, see: Becker *et al.* (1987 ▶); Seliger *et al.* (1990 ▶); Sugawara *et al.* (1999 ▶); Tezer & Karakus (2009 ▶). For bond-length data, see: Allen *et al.* (1987 ▶); Ogawa & Harada (2003 ▶).
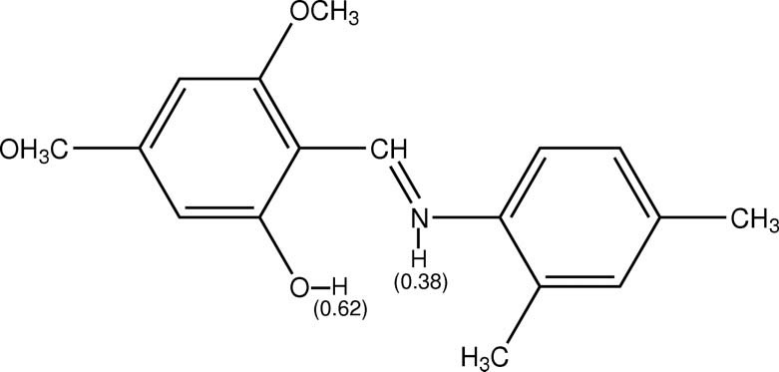


## Experimental

### 

#### Crystal data

C_17_H_19_NO_3_
*M* *_r_* = 285.33
Monoclinic, 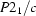

*a* = 4.7070 (2) Å
*b* = 11.283 (5) Å
*c* = 28.216 (5) Å
β = 97.542 (11)°
*V* = 1485.6 (7) Å^3^
*Z* = 4
Mo *K*α radiation
μ = 0.09 mm^−1^
*T* = 296 K
0.67 × 0.31 × 0.09 mm

#### Data collection

Stoe IPDS II diffractometer
Absorption correction: integration (*X-RED32*; Stoe & Cie, 2002 ▶) *T*_min_ = 0.977, *T*_max_ = 0.994
13316 measured reflections
2797 independent reflections
1808 reflections with *I* > 2σ(*I*)
*R*_int_ = 0.043

#### Refinement

*R*[*F* ^2^ > 2σ(*F* ^2^)] = 0.043
*wR*(*F* ^2^) = 0.111
*S* = 0.98
2797 reflections
195 parameters
H-atom parameters constrained
Δρ_max_ = 0.09 e Å^−3^
Δρ_min_ = −0.14 e Å^−3^

### 

Data collection: *X-AREA* (Stoe & Cie, 2002 ▶); cell refinement: *X-AREA*; data reduction: *X-RED32*; program(s) used to solve structure: *SHELXS97* (Sheldrick, 2008 ▶); program(s) used to refine structure: *SHELXL97* (Sheldrick, 2008 ▶); molecular graphics: *ORTEP-3 for Windows* (Farrugia, 1997 ▶); software used to prepare material for publication: *WinGX* (Farrugia, 1999 ▶).

## Supplementary Material

Crystal structure: contains datablocks I, global. DOI: 10.1107/S1600536809021278/ci2808sup1.cif

Structure factors: contains datablocks I. DOI: 10.1107/S1600536809021278/ci2808Isup2.hkl

Additional supplementary materials:  crystallographic information; 3D view; checkCIF report

## Figures and Tables

**Table 1:** Hydrogen-bond geometry (Å, °)

*D*—H⋯*A*	*D*—H	H⋯*A*	*D*⋯*A*	*D*—H⋯*A*
O1—H1*A*⋯N1	0.82	1.82 (1)	2.561 (2)	149
N1—H1*B*⋯O1	0.86	1.86 (1)	2.561 (2)	137 (1)

**Fig. 1.**
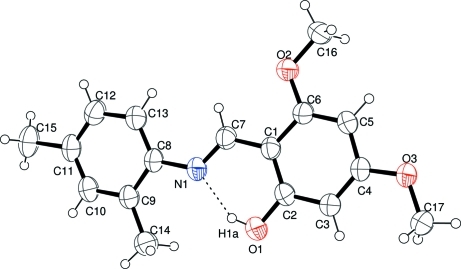
The molecular structure of the title compound, showing the atom-numbering scheme and 50% probability diplacement ellipsoids. Only the enol (OH) tautomer is shown. Also, only one component of the disordered C15-methyl group is shown.

## supplementary crystallographic information

### Comment

Proton tautomerism plays an important role in many fields of chemistry and biochemistry (Sugawara *et al.*, 1999). The study of ground state intramolecular proton (hydrogen) transfer (IPT) reactions have received increasing attention in recently aiming at the characterization of a large number of compounds in which rapid hydrogen migration occurs both in solution and in solid state. Salicilidine aniline and its derivatives are among the earliest examples of a chemical system involving IPT. A prototropic tautomeric attitude has been recognized in a number of aromatic Schiff bases (Becker *et al.*, 1987; Seliger *et al.*, 1990; Tezer & Karakus, 2009). *O*-hydroxy Schiff bases are of interest mainly due to the existence of O—H···N and N—H···O type hydrogen bonds and tautomerism between the enol-imine (OH) and keto-amine (NH) forms.

The X-ray analysis reveals that in the title compound both the enol (OH) and keto (NH) tautomers coexist with occupancies of 0.62 (3) and 0.38 (3), respectively. But we are unable to confirm the presence of minor keto form from the IR spectrum. The major enol (OH) tautomer is shown in Fig. 1. The C2—O1 [1.3312 (18) Å] bond length is intermediate between the C—O single (1.362 Å) and C═O double (1.222 Å) bonds. Similarly, the C7—N1 bond length of 1.293 (2) Å is shorter than C—N and C═N bond lengths (1.339 and 1.279 Å, respectively; Allen *et al.*, 1987). The shortened C2—O1 bond and the slightly longer C7—N1 bond provide structural evidence for the double tautomeric forms of the title compound. Similar result was observed for *N*-(5-chloro-2-hydroxybenzylidene) -4-hydroxyaniline [C—O = 1.321 (2) and C—N = 1.293 (2) Å; Ogawa & Harada, 2003].

The molecule of the title compound is approximately planar, with the dihedral angle between the two aromatic rings being 6.97 (8)°. The molecular structure is stabilized by an intramolecular O1—H1A···N1 hydrogen bond (Table 1) which generates a six-membered ring, producing a S(6) ring motif. It is known that Schiff bases may exhibit thermochromism or photochromism, depending on the planarity or non-planarity of the molecule, respectively. Therefore, the title compound may exhibit thermochromic properties.

No significant π-π interactions are observed in the crystal structure.

### Experimental

A solution of 2-hydroxy-4,6-dimethoxy-benzaldehyde (0.0389 g, 0.21 mmol) in ethanol (20 ml) was added to a solution of 2,4-dimethylaniline (0.0263 g, 0.21 mmol) in ethanol (20 ml). The reaction mixture was stirred for 1 h under reflux. Single crystals suitable for X-ray analysis were obtained from ethyl alcohol by slow evaporation (yield 59%; m.p.386–388 K).

### Refinement

C-bound H atoms were positioned geometrically and refined using a riding model, with C-H = 0.93-0.96 Å and *U*_iso_(H) = 1.2-1.5*U*_eq_(C). The C15-methyl group was refined as idealized disordered one with two positions rotated from each other by 60°. At this stage, the hydroxyl H atom, H1A, was located in a difference map and attempts to refine it freely resulted in a very long O-H distance of 1.22 (3) Å. When the O-bound H atom was positioned geometrically a new peak at 0.97 Å from N1 was observed, indicating a disorder in the H atom i.e the presence of both OH and NH tautomers. At this stage both O and N-bound H atoms, H1A and H1B, were positioned geometrically and their occupancies were refined to 0.62 (3) and 0.38 (3), respectively. The refinement shows the enol form is the major component. The IR spectrum of the compound confirms only the presence of enol form and we are unable to confirm the minor keto form from the IR spectrum.

### Crystal data

**Table:**

C_17_H_19_NO_3_	*F*(000) = 608
*M**_r_* = 285.33	*D*_x_ = 1.276 Mg m^−^^3^
Monoclinic, *P*2_1_/*c*	Mo *K*α radiation, λ = 0.71069 Å
Hall symbol: -P 2ybc	Cell parameters from 10962 reflections
*a* = 4.7070 (2) Å	θ = 1.5–26.2°
*b* = 11.283 (5) Å	µ = 0.09 mm^−^^1^
*c* = 28.216 (5) Å	*T* = 296 K
β = 97.542 (11)°	Prism, yellow
*V* = 1485.6 (7) Å^3^	0.67 × 0.31 × 0.09 mm
*Z* = 4	

### Data collection

**Table:**

Stoe IPDS II diffractometer	2797 independent reflections
Radiation source: fine-focus sealed tube	1808 reflections with *I* > 2σ(*I*)
plane graphite	*R*_int_ = 0.043
Detector resolution: 6.67 pixels mm^-1^	θ_max_ = 25.6°, θ_min_ = 1.5°
rotation method scans	*h* = −5→5
Absorption correction: integration (*X-RED32*; Stoe & Cie, 2002)	*k* = −13→13
*T*_min_ = 0.977, *T*_max_ = 0.994	*l* = −34→34
13316 measured reflections	

### Refinement

**Table:**

Refinement on *F*^2^	Primary atom site location: structure-invariant direct methods
Least-squares matrix: full	Secondary atom site location: difference Fourier map
*R*[*F*^2^ > 2σ(*F*^2^)] = 0.043	Hydrogen site location: inferred from neighbouring sites
*wR*(*F*^2^) = 0.111	H-atom parameters constrained
*S* = 0.98	*w* = 1/[σ^2^(*F*_o_^2^) + (0.0599*P*)^2^] where *P* = (*F*_o_^2^ + 2*F*_c_^2^)/3
2797 reflections	(Δ/σ)_max_ = 0.001
195 parameters	Δρ_max_ = 0.09 e Å^−^^3^
0 restraints	Δρ_min_ = −0.14 e Å^−^^3^

### Special details

**Table:**

Experimental. 240 frames, detector distance = 130 mm
Geometry. All e.s.d.'s (except the e.s.d. in the dihedral angle between two l.s. planes) are estimated using the full covariance matrix. The cell e.s.d.'s are taken into account individually in the estimation of e.s.d.'s in distances, angles and torsion angles; correlations between e.s.d.'s in cell parameters are only used when they are defined by crystal symmetry. An approximate (isotropic) treatment of cell e.s.d.'s is used for estimating e.s.d.'s involving l.s. planes.
Refinement. Refinement of *F*^2^ against ALL reflections. The weighted *R*-factor *wR* and goodness of fit *S* are based on *F*^2^, conventional *R*-factors *R* are based on *F*, with *F* set to zero for negative *F*^2^. The threshold expression of *F*^2^ > σ(*F*^2^) is used only for calculating *R*-factors(gt) *etc*. and is not relevant to the choice of reflections for refinement. *R*-factors based on *F*^2^ are statistically about twice as large as those based on *F*, and *R*- factors based on ALL data will be even larger.

### Fractional atomic coordinates and isotropic or equivalent isotropic displacement parameters (Å^2^)

**Table:**

	*x*	*y*	*z*	*U*_iso_*/*U*_eq_	Occ. (<1)
O1	−0.1970 (3)	0.07461 (10)	0.06735 (5)	0.0759 (4)	
H1A	−0.0686	0.0847	0.0896	0.114*	0.62 (3)
O2	−0.3588 (3)	0.47471 (10)	0.10480 (4)	0.0735 (4)	
O3	−0.9431 (3)	0.29177 (10)	−0.02381 (4)	0.0721 (4)	
N1	0.1167 (3)	0.18286 (12)	0.13482 (5)	0.0584 (4)	
H1B	0.0796	0.1207	0.1175	0.070*	0.38 (3)
C1	−0.2694 (3)	0.27691 (13)	0.08638 (6)	0.0534 (4)	
C2	−0.3428 (4)	0.17509 (14)	0.05865 (6)	0.0580 (4)	
C3	−0.5703 (4)	0.17634 (14)	0.02150 (6)	0.0610 (5)	
H3	−0.6186	0.1085	0.0035	0.073*	
C4	−0.7208 (3)	0.27934 (14)	0.01202 (6)	0.0569 (4)	
C5	−0.6564 (4)	0.38249 (14)	0.03878 (6)	0.0593 (4)	
H5	−0.7616	0.4515	0.0317	0.071*	
C6	−0.4362 (3)	0.38028 (13)	0.07557 (6)	0.0553 (4)	
C7	−0.0392 (3)	0.27603 (14)	0.12467 (6)	0.0565 (4)	
H7	−0.0002	0.3443	0.1428	0.068*	
C8	0.3450 (3)	0.17699 (14)	0.17280 (6)	0.0555 (4)	
C9	0.4693 (3)	0.06605 (15)	0.18298 (6)	0.0590 (4)	
C10	0.6896 (4)	0.05720 (16)	0.22082 (6)	0.0657 (5)	
H10	0.7713	−0.0168	0.2279	0.079*	
C11	0.7925 (4)	0.15256 (17)	0.24829 (6)	0.0655 (5)	
C12	0.6723 (4)	0.26180 (17)	0.23629 (7)	0.0694 (5)	
H12	0.7412	0.3283	0.2536	0.083*	
C13	0.4512 (4)	0.27460 (16)	0.19910 (6)	0.0661 (5)	
H13	0.3736	0.3492	0.1918	0.079*	
C14	0.3707 (4)	−0.04062 (15)	0.15315 (7)	0.0758 (6)	
H14A	0.4782	−0.1089	0.1653	0.114*	
H14B	0.4005	−0.0269	0.1206	0.114*	
H14C	0.1706	−0.0540	0.1546	0.114*	
C15	1.0290 (4)	0.1388 (2)	0.28958 (7)	0.0865 (6)	
H15A	0.9977	0.1930	0.3146	0.130*	0.50
H15B	1.2103	0.1555	0.2789	0.130*	0.50
H15C	1.0293	0.0590	0.3015	0.130*	0.50
H15D	1.1605	0.0787	0.2821	0.130*	0.50
H15E	0.9479	0.1162	0.3177	0.130*	0.50
H15F	1.1289	0.2127	0.2952	0.130*	0.50
C16	−0.5298 (4)	0.57876 (15)	0.09754 (7)	0.0788 (6)	
H16A	−0.4572	0.6380	0.1204	0.118*	
H16B	−0.7242	0.5602	0.1015	0.118*	
H16C	−0.5232	0.6082	0.0658	0.118*	
C17	−1.0150 (5)	0.19050 (17)	−0.05323 (8)	0.0852 (7)	
H17A	−1.1735	0.2093	−0.0770	0.128*	
H17B	−1.0670	0.1261	−0.0339	0.128*	
H17C	−0.8529	0.1679	−0.0686	0.128*	

### Atomic displacement parameters (Å^2^)

**Table:**

	*U*^11^	*U*^22^	*U*^33^	*U*^12^	*U*^13^	*U*^23^
O1	0.0732 (8)	0.0612 (7)	0.0852 (9)	0.0155 (6)	−0.0198 (7)	−0.0036 (7)
O2	0.0791 (9)	0.0578 (7)	0.0746 (8)	0.0127 (6)	−0.0235 (7)	−0.0093 (6)
O3	0.0703 (8)	0.0676 (7)	0.0697 (8)	0.0140 (6)	−0.0237 (6)	−0.0097 (6)
N1	0.0537 (8)	0.0624 (8)	0.0559 (8)	0.0062 (6)	−0.0046 (7)	0.0033 (6)
C1	0.0488 (9)	0.0570 (9)	0.0529 (9)	0.0024 (7)	0.0002 (7)	0.0055 (7)
C2	0.0541 (10)	0.0562 (9)	0.0613 (10)	0.0076 (7)	−0.0010 (8)	0.0058 (8)
C3	0.0585 (10)	0.0574 (9)	0.0635 (10)	0.0047 (8)	−0.0058 (8)	−0.0043 (8)
C4	0.0513 (9)	0.0615 (9)	0.0548 (10)	0.0041 (7)	−0.0048 (8)	0.0020 (8)
C5	0.0575 (10)	0.0553 (9)	0.0616 (10)	0.0094 (7)	−0.0054 (8)	0.0037 (8)
C6	0.0555 (10)	0.0538 (8)	0.0545 (9)	0.0004 (7)	−0.0009 (8)	0.0006 (7)
C7	0.0534 (9)	0.0598 (9)	0.0549 (10)	0.0010 (8)	0.0009 (8)	0.0021 (7)
C8	0.0476 (9)	0.0671 (10)	0.0499 (9)	0.0022 (7)	−0.0013 (7)	0.0033 (8)
C9	0.0538 (10)	0.0672 (10)	0.0542 (10)	0.0052 (8)	0.0008 (8)	0.0053 (8)
C10	0.0570 (10)	0.0762 (11)	0.0614 (11)	0.0099 (8)	−0.0018 (9)	0.0105 (9)
C11	0.0494 (10)	0.0920 (13)	0.0531 (10)	0.0037 (9)	−0.0002 (8)	0.0085 (9)
C12	0.0607 (11)	0.0834 (12)	0.0613 (11)	−0.0051 (9)	−0.0023 (9)	−0.0070 (9)
C13	0.0595 (11)	0.0696 (10)	0.0661 (11)	0.0056 (8)	−0.0034 (9)	−0.0001 (9)
C14	0.0810 (13)	0.0660 (11)	0.0750 (12)	0.0091 (9)	−0.0097 (10)	0.0030 (9)
C15	0.0623 (12)	0.1271 (17)	0.0645 (12)	−0.0006 (11)	−0.0125 (10)	0.0102 (12)
C16	0.0879 (14)	0.0586 (10)	0.0823 (13)	0.0180 (9)	−0.0175 (11)	−0.0103 (9)
C17	0.0864 (14)	0.0770 (12)	0.0816 (13)	0.0157 (10)	−0.0293 (11)	−0.0214 (10)

### Geometric parameters (Å, °)

**Table:**

O1—C2	1.3312 (18)	C10—C11	1.376 (3)
O1—H1A	0.82	C10—H10	0.93
O2—C6	1.3673 (19)	C11—C12	1.380 (3)
O2—C16	1.4229 (19)	C11—C15	1.510 (2)
O3—C4	1.3634 (18)	C12—C13	1.385 (2)
O3—C17	1.427 (2)	C12—H12	0.93
N1—C7	1.293 (2)	C13—H13	0.93
N1—C8	1.4159 (19)	C14—H14A	0.96
N1—H1B	0.86	C14—H14B	0.96
C1—C2	1.407 (2)	C14—H14C	0.96
C1—C6	1.416 (2)	C15—H15A	0.96
C1—C7	1.426 (2)	C15—H15B	0.96
C2—C3	1.397 (2)	C15—H15C	0.96
C3—C4	1.369 (2)	C15—H15D	0.96
C3—H3	0.93	C15—H15E	0.96
C4—C5	1.399 (2)	C15—H15F	0.96
C5—C6	1.367 (2)	C16—H16A	0.96
C5—H5	0.93	C16—H16B	0.96
C7—H7	0.93	C16—H16C	0.96
C8—C13	1.384 (2)	C17—H17A	0.96
C8—C9	1.396 (2)	C17—H17B	0.96
C9—C10	1.391 (2)	C17—H17C	0.96
C9—C14	1.507 (2)		
			
C2—O1—H1A	109.5	C8—C13—C12	120.38 (17)
C6—O2—C16	117.08 (12)	C8—C13—H13	119.8
C4—O3—C17	116.70 (13)	C12—C13—H13	119.8
C7—N1—C8	123.93 (15)	C9—C14—H14A	109.5
C7—N1—H1B	118.0	C9—C14—H14B	109.5
C8—N1—H1B	118.0	H14A—C14—H14B	109.5
C2—C1—C6	117.64 (14)	C9—C14—H14C	109.5
C2—C1—C7	121.47 (14)	H14A—C14—H14C	109.5
C6—C1—C7	120.88 (14)	H14B—C14—H14C	109.5
O1—C2—C3	118.22 (14)	C11—C15—H15A	109.5
O1—C2—C1	120.65 (14)	C11—C15—H15B	109.5
C3—C2—C1	121.13 (14)	H15A—C15—H15B	109.5
C4—C3—C2	118.77 (15)	C11—C15—H15C	109.5
C4—C3—H3	120.6	H15A—C15—H15C	109.5
C2—C3—H3	120.6	H15B—C15—H15C	109.5
O3—C4—C3	124.01 (14)	C11—C15—H15D	109.5
O3—C4—C5	113.93 (13)	H15A—C15—H15D	141.1
C3—C4—C5	122.06 (14)	H15B—C15—H15D	56.3
C6—C5—C4	118.92 (14)	H15C—C15—H15D	56.3
C6—C5—H5	120.5	C11—C15—H15E	109.5
C4—C5—H5	120.5	H15A—C15—H15E	56.3
O2—C6—C5	124.00 (14)	H15B—C15—H15E	141.1
O2—C6—C1	114.55 (13)	H15C—C15—H15E	56.3
C5—C6—C1	121.46 (15)	H15D—C15—H15E	109.5
N1—C7—C1	121.77 (15)	C11—C15—H15F	109.5
N1—C7—H7	119.1	H15A—C15—H15F	56.3
C1—C7—H7	119.1	H15B—C15—H15F	56.3
C13—C8—C9	119.42 (15)	H15C—C15—H15F	141.1
C13—C8—N1	123.59 (15)	H15D—C15—H15F	109.5
C9—C8—N1	116.99 (14)	H15E—C15—H15F	109.5
C10—C9—C8	118.20 (16)	O2—C16—H16A	109.5
C10—C9—C14	121.02 (15)	O2—C16—H16B	109.5
C8—C9—C14	120.76 (14)	H16A—C16—H16B	109.5
C11—C10—C9	123.23 (17)	O2—C16—H16C	109.5
C11—C10—H10	118.4	H16A—C16—H16C	109.5
C9—C10—H10	118.4	H16B—C16—H16C	109.5
C10—C11—C12	117.21 (16)	O3—C17—H17A	109.5
C10—C11—C15	121.56 (18)	O3—C17—H17B	109.5
C12—C11—C15	121.23 (18)	H17A—C17—H17B	109.5
C11—C12—C13	121.49 (17)	O3—C17—H17C	109.5
C11—C12—H12	119.3	H17A—C17—H17C	109.5
C13—C12—H12	119.3	H17B—C17—H17C	109.5
			
C6—C1—C2—O1	−179.15 (16)	C7—C1—C6—C5	179.61 (16)
C7—C1—C2—O1	−0.4 (3)	C8—N1—C7—C1	−179.15 (15)
C6—C1—C2—C3	0.6 (3)	C2—C1—C7—N1	1.8 (3)
C7—C1—C2—C3	179.39 (17)	C6—C1—C7—N1	−179.44 (15)
O1—C2—C3—C4	−179.62 (16)	C7—N1—C8—C13	−9.1 (3)
C1—C2—C3—C4	0.6 (3)	C7—N1—C8—C9	172.11 (16)
C17—O3—C4—C3	−1.3 (3)	C13—C8—C9—C10	2.6 (3)
C17—O3—C4—C5	178.46 (17)	N1—C8—C9—C10	−178.54 (15)
C2—C3—C4—O3	178.77 (17)	C13—C8—C9—C14	−176.31 (17)
C2—C3—C4—C5	−0.9 (3)	N1—C8—C9—C14	2.6 (2)
O3—C4—C5—C6	−179.76 (16)	C8—C9—C10—C11	−0.7 (3)
C3—C4—C5—C6	0.0 (3)	C14—C9—C10—C11	178.17 (18)
C16—O2—C6—C5	3.4 (3)	C9—C10—C11—C12	−1.5 (3)
C16—O2—C6—C1	−176.22 (17)	C9—C10—C11—C15	179.01 (18)
C4—C5—C6—O2	−178.32 (16)	C10—C11—C12—C13	1.9 (3)
C4—C5—C6—C1	1.3 (3)	C15—C11—C12—C13	−178.59 (18)
C2—C1—C6—O2	178.09 (15)	C9—C8—C13—C12	−2.2 (3)
C7—C1—C6—O2	−0.7 (2)	N1—C8—C13—C12	178.98 (17)
C2—C1—C6—C5	−1.6 (3)	C11—C12—C13—C8	−0.1 (3)

### Hydrogen-bond geometry (Å, °)

**Table:**

*D*—H···*A*	*D*—H	H···*A*	*D*···*A*	*D*—H···*A*
O1—H1A···N1	0.82	1.82 (1)	2.561 (2)	149
N1—H1B···O1	0.86	1.86 (1)	2.561 (2)	137 (1)
